# Slit2/Robo1 signaling constrains image stabilization responses to preserve ethologically favorable directional asymmetry

**DOI:** 10.1016/j.cub.2025.08.030

**Published:** 2025-09-12

**Authors:** James K. Kiraly, Annika Balraj, Paige Leary, Zihao You, Scott C. Harris, Jeanette D. Hyer, Felice A. Dunn, Alex L. Kolodkin

**Affiliations:** 1 Solomon H. Snyder Department of Neuroscience, The Johns Hopkins Kavli Neuroscience Discovery Institute, The Johns Hopkins University School of Medicine, Baltimore, MD 21205, USA; 2 Department of Ophthalmology, University of California, San Francisco, San Francisco, CA 94158, USA; 3 Lead contact

## Abstract

Visual sensation relies on retinal circuitry to receive environmental inputs and convey relevant information for behavioral outputs. Many species depend on gaze stabilization behaviors, such as the optokinetic reflex (OKR), to perceive and correct for global motion. OKR calculation begins in the retina, where ON direction-selective ganglion cells (oDSGCs) respond to slow visual motion and deliver information to the accessory optic system (AOS) to inform oculomotor outputs. Here, we find that the guidance receptor roundabout-1 (*Robo1*) and its ligand *Slit2* are selectively expressed in mammalian retinal AOS components and refine oDSGC circuitry to constrain OKR gain for appropriate image stabilization responses. *Robo1* or *Slit2* loss-of-function (LOF) mutants exhibit an increase in OKR gain, reducing directional asymmetry by elevating naturally weaker OKR responses. These behavioral enhancements largely arise from alterations in the retina, and loss of *Slit2* in starburst amacrine cells (SACs) enhances oDSGC firing properties, with increased spike responses, excitatory synaptic puncta, and downstream AOS activation. These findings reveal that the Slit2/Robo1 signaling tunes oDSGC circuitry to maintain regulated image stabilization behavior.

## In brief

Kiraly et al. show that Slit2/Robo1 signaling refines ON direction-selective ganglion cell synapses to constrain the optokinetic reflex and conserve image tracking asymmetries commensurate with balancing optic flow biases during self-motion. These findings reveal how retinal motion detection favorably influences the magnitude of eye movements for ethological relevance.

## Graphical Abstract

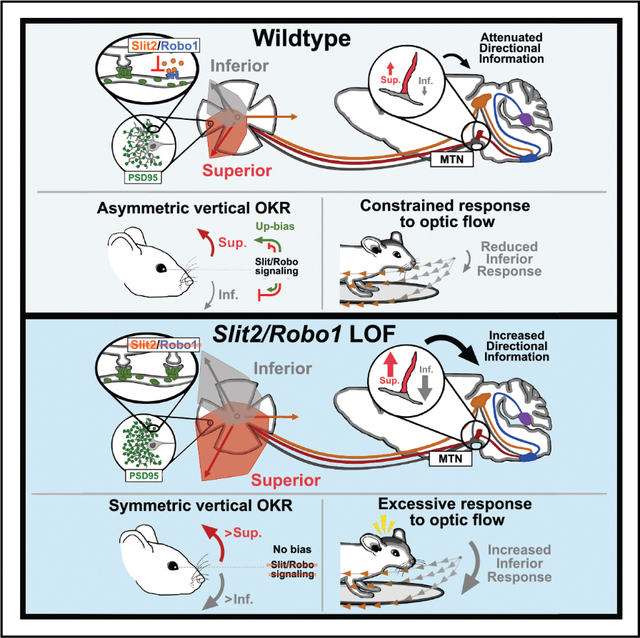

## INTRODUCTION

Many animals rely on visual motion perception in dynamic environments, and gaze stabilization behaviors are required to compensate for global optic flow and maintain images within the visual field. The optokinetic reflex (OKR) is an oculomotor response induced by slow motion to reduce retinal slip through reflexive tracking along the directional axis of motion accompanied by opposing compensatory saccades.^1–3^ The OKR is an ancient visual response conserved within the animal kingdom across a variety of environments, functioning with the vestibulo-ocular reflex (VOR) to stabilize images via ocular and head rotation.^1,2,4–8^ Optokinetic responses exhibit conserved directional asymmetries, with a bias toward ventro-dorsal (superior) motion and temporo-nasal (forward) motion and against responses to dorso-ventral (inferior) and naso-temporal (backward) motion.^1,9–12^ These asymmetries, thought to balance the dominant optic flow patterns of forward movement near the ground, lead to the hypothesis that they prioritize ethologically relevant directional optic flow and attenuate less consequential visual information.^1^

The OKR is initiated by retinal direction-selective (DS) circuits, which convey retinal slip information for visuovestibular processing.^13,14^ To calculate directionality, retinal ganglion cells known as ON DS ganglion cells (oDSGCs) are tuned to respond to slow motion in distinct directions. DS in oDSGCs depends on inhibitory input from ON starburst amacrine cells (SACs), which are morphologically radially symmetric, cholinergic/GABAergic interneurons that encode directional motion. Through integration of SAC-derived inhibition with excitatory inputs, oDSGCs spike selectively to directional motion across the retina, generating a tuning curve along which they maximally or minimally respond.^9,13–16^ oDSGCs are organized along the axes of the semicircular canals and include three primary subtypes: superior, inferior, and forward oDSGCs, preferentially responding to upward, downward, and temporo-nasal motion in the visual field, respectively.^15^ Recent evidence includes a rare oDSGC subtype that responds nasotemporally.^17^ These oDSGC tuning properties, both in directionality and magnitude, direct the computation that generates image-stabilizing eye movements.^9^

To convey vertical motion, superior and inferior oDSGCs project to the medial terminal nucleus (MTN) and the lateral terminal nucleus (LTN), respectively.^4,18–21^ In mice, the LTN is integrated into the MTN, dividing the MTN into dorsal and ventral (dMTN and vMTN) subnuclei that encode upward and downward motion, respectively.^9,18,20^ Forward oDSGCs project to the nucleus of the optic tract and the dorsal terminal nucleus (NOT/DTN) to encode horizontal motion.^18,21,22^ From oDSGC input, directional information is sent through the accessory optic system (AOS) to the inferior olivary complex and ultimately the cerebellar flocculus to regulate oculomotor output.^18^

Previous work describes the generation of select oDSGC subtypes^23^ and establishment of connectivity within the retina^16,24^ and to the brain.^19,25^ Further, vertical OKR asymmetry is linked to retinal oDSGC tuning properties, rather than central processing.^9^ However, little is known about the specific mechanisms that refine oDSGC synaptic properties necessary to establish retinal control over vertical OKR gain.

Here, we show that the roundabout-1 (Robo1) guidance receptor and its canonical ligand Slit2 constrain OKR responses in the retina to generate regulated behavioral outputs through refinement of oDSGC circuitry. Robo1 and the related Robo2 receptors are the only Robo family members robustly expressed in the mammalian retina, and Robo2 plays a major role in visual system development.^26–29^ We find that Robo1 serves two additional roles in oDSGCs: shaping oDSGC synaptic properties in the retina through SAC-derived ligand Slit2 and refining oDSGC axonal projections to the MTN. These observations advance our understanding of how oDSGC tuning generates naturalistic image stabilization behaviors and demonstrate that Slit/Robo signaling is critical for controlling the AOS-mediated vertical OKR asymmetry.

## RESULTS

### *Robo1* is highly and specifically expressed in AOS oDSGCs

**Figure 1.**
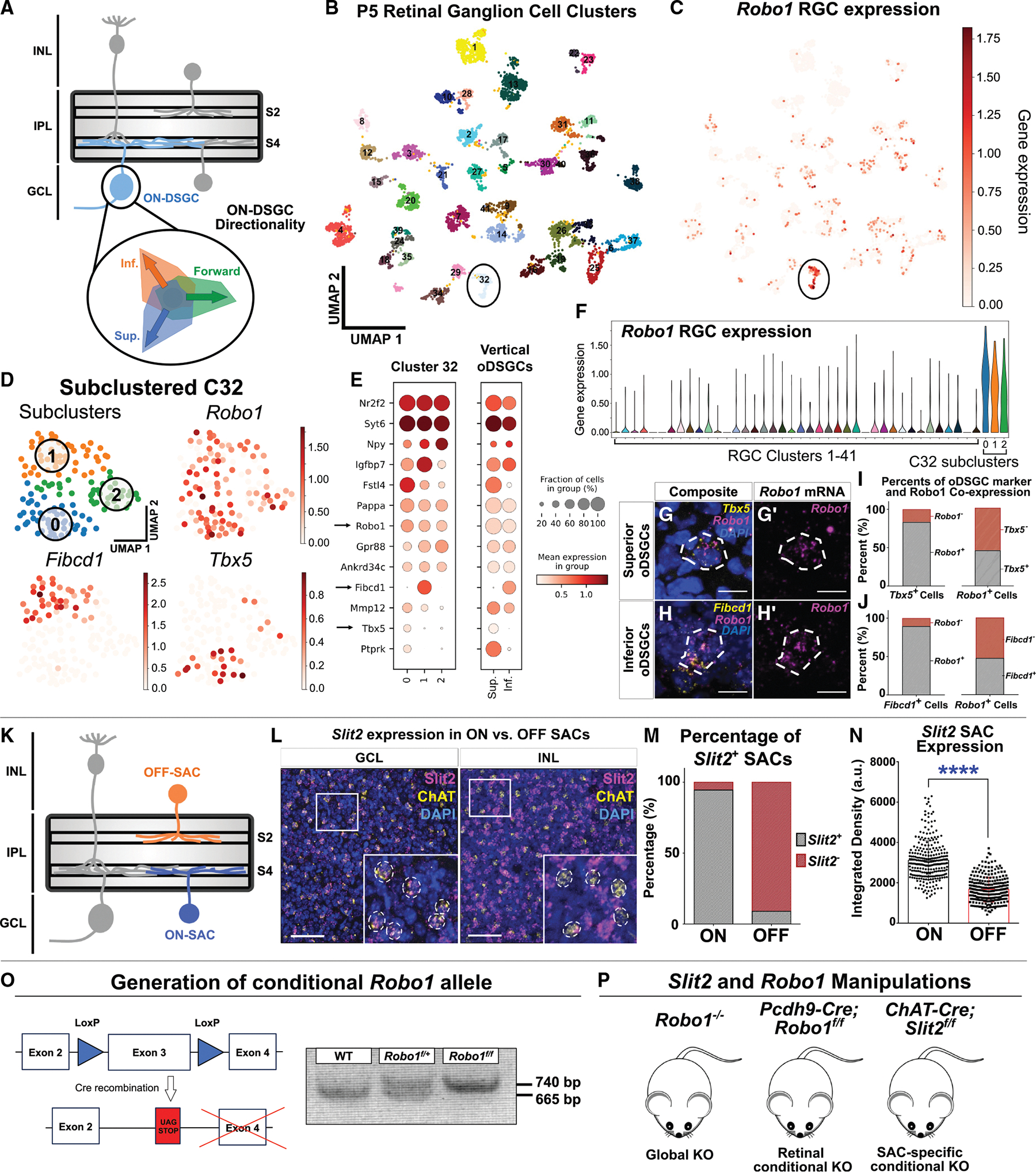
*Robo1* and *Slit2* are highly enriched in DS circuitry (A) oDSGCs exist in three main types: inferior, superior, and forward. (B) Uniform manifold approximation and projection (UMAP) plot of neonatal RGC clusters described previously.^29^ AOS oDSGCs reside in cluster 32 (C32).^22^ (C) UMAP plot of *Robo1* gene expression in RGCs. (D) Unsupervised sub-clustering of C32 reveals three distinct subclusters. Subclusters 0 and 1 express the superior oDSGC marker *Tbx5* and the inferior oDSGC marker *Fibcd1*, respectively. *Robo1* is highly enriched in all three oDSGC clusters. (E) Dotplots of C32 and vertical oDSGC marker expression in subclusters 0, 1, and 2 of C32, and also in vertical oDSGCs.^23^ Note that C32 subclusters express appropriate vertical oDSGC markers (arrows) and that vertical oDSGCs express C32 markers. (F) Violin plot of *Robo1* expression in C32 subclusters. (G and H) HCR *in situ* hybridization confirms *Robo1* co-expression in superior *Tbx5*^+^ (G) and inferior *Fibcd1*^+^ (H) oDSGCs. (I) Quantification of *Tbx5*^+^ and *Robo1*^+^ and *Tbx5*^−^ but *Robo1*^+^ cells within the same sections. (J) Quantification of *Fibcd1*^+^ and *Robo1*^+^ and *Fibcd1*^−^ but *Robo1*^+^ cells. Expression is defined as cells with >5 fluorescent puncta. (K) SACs exist as ON or OFF types. ON-SACs directly contact oDSGCs. (L) HCR *in situ* hybridization of *Slit2* expression co-localized with *ChAT* in SACs. (M and N) ON-SACs express *Slit2* at levels greater than OFF-SACs. Expression is defined as *ChAT* ^+^ cells with >5 *Slit2* puncta (M) or by fluorescent intensity of cells (N). (O) Schematic showing generation of *Robo1*^*f/f*^ allele. (P) Lines used to interrogate *Slit2* and *Robo1* function. Scale bars, 10 μm (G and H) and 500 μm (L). ****p < 0.0001. Bar graphs and error bars indicate the mean and standard deviation (SD). See also Figure S1.

To understand the oDSGC refinement necessary to control OKR output, we investigated signaling pathway components specifically expressed in oDSGCs (Figure 1A). We analyzed a transcriptomic atlas of P5 RGCs^30^ for genes selectively expressed during oDSGC refinement (Figure 1B). In this atlas, oDSGCs reside in cluster 32 (C32), a cluster that expresses specific markers for both superior and inferior oDSGCs^23^ in addition to other known oDSGC markers^31–33^ (Figure 1B). We examined C32 for oDSGC-specific genes and found selective expression of *Robo1* in oDSGCs (Figure 1C). To confirm cell identities, we performed unsupervised sub-clustering of C32 (STAR Methods) and found three subclusters: one that expresses the specific superior oDSGC marker *Tbx5*,^23^ another that expresses the specific inferior oDSGC marker *Fibcd1*,^23^ and a third that has not been described (Figure 1D). Since oDSGCs exist primarily in three major types,^15,21^ it is likely these three subclusters correspond to the three oDSGC types: *Tbx5*^+^ superior, *Fibcd1*^+^ inferior, and a putative forward oDSGC type. Using a transcriptomic profile of superior and inferior cells,^23^ we found (1) oDSGC type-specific markers are expressed in C32 subclusters, and (2) several C32-specific markers are expressed in vertically tuned oDSGCs (Figure 1E). *Robo1* expression is specific to C32, is observed in all three oDSGC types, and is not strong in any other RGC types (Figures 1D–1F; putative “forward” cluster: “2” in Figure 1E). Since only vertically tuned oDSGC markers are known,^23^ we confirmed that vertically tuned oDSGCs express *Robo1* at post-natal day 6 (P6) through hybridization chain reaction (HCR) fluorescent *in situ* hybridizations of *Robo1* co-localized with the superior marker *Tbx5* (Figure 1G) and the inferior marker *Fibcd1* (Figure 1H). *Robo1* co-localization with these markers demonstrates that nearly all vertically tuned oDSGCs express *Robo1* and that this expression comprises most RGC *Robo1* expression (Figures 1I and 1J). These data establish that *Robo1* is highly and specifically expressed in all three murine oDSGC directional types.

### Specificity of *Robo1* mRNA expression in oDSGCs is conserved across mammalian species, including humans

OKR asymmetries are conserved across species, so we asked whether oDSGC-specific *Robo1* expression is similarly conserved in mammals. We analyzed two RGC transcriptomic atlases: an integrated dataset of 15 mammalian species^33^ and a dataset of human fetal retina across development.^34^ In this cross-species RGC dataset,^33^ ortholog cluster 19 was described previously as containing AOS oDSGCs due to expression of AOS markers, including *BNC2*, a marker for oDSGCs in primates and humans^31^ (Figure S1B), and also expression of known murine oDSGC marker genes^30^ such as *PAPPA* and *SYT6*^30,33^ (Figures S1B and S1C). We found *Robo1* among ortholog cluster 19 markers, with high levels of expression and conservation across mammalian RGC transcriptomes.

Analysis of the human fetal RGC transcriptome confirms high levels of *ROBO1* expression in putative human oDSGCs. Unsupervised clustering of human RGCs integrated across developmental time reveals a small cluster, cluster 21, as a putative oDSGC cluster due to its expression profile (Figures S1D and S1E). *ROBO1*, along with a novel *ROBO1*-associated lncRNA, are highly enriched in cluster 21 and are the top differentially expressed marker genes in this putative oDSGC cluster (Figures S1F–S1H). Expression of *ROBO1* during human embryonic development mirrors expression in the embryonic mouse retina, where *Robo1* exhibits high specificity in oDSGCs even at E13.^35^ These data demonstrate that *Robo1* specificity in oDSGCs is conserved in the mammalian retina, suggesting shared functions in AOS circuitry.

### Canonical Robo ligand *Slit2* is differentially expressed in ON-SACs compared with OFF-SACs

We next investigated expression of Robo ligands in SACs to determine if they function together with Robo1 in DS circuit elaboration and function (Figure 1K). Canonical Robo receptor signaling involves secreted Slit protein ligands to direct axon pathfinding, dendritic morphology, and synapse formation.^26,27,29,36–39^ *Slit2* is differentially expressed between ON- and OFF-SACs, with significantly higher enrichment in ON-SACs, which directly interface with oDSGCs.^40,41^ We confirmed these results through HCR fluorescent *in situ* hybridization, detecting *Slit2* co-localization with the SAC marker *ChAT* and observing significantly higher levels of *Slit2* in ON-SACs compared with OFF-SACs (Figures 1L–1N) despite high levels of *Slit2* in other INL cell types, including OFF bipolar cells.^42^ Since oDSGC-SAC interactions are critical for DS responses,^13,14^ the specificity of *Slit2*/*Robo1* expression suggested that SAC-derived Slit2 signaling via Robo1 expressed in oDSGC shapes DS circuits.

### Disruption of *Robo1* or *Slit2* enhances OKR tracking responses to vertical and horizontal directional motion

We next generated a novel *Robo1* conditional knockout line (Figure 1O), which, along with existing *Robo1* null and *Slit2* conditional alleles^36,43–45^ (Figure 1P), allowed us to assess the impact of *Slit2* or *Robo1* loss on DS circuitry. We used the *Robo1* global knockout (*Robo1*^−/−^),^36^ the retinal *Robo1* conditional knockout (*Pcdh9-Cre; Robo1*^*f/f*^),^45^ and the SAC-specific *Slit2* conditional knockout (*ChAT-Cre; Slit2*^*f/f*^)^43,44^ animals for these experiments. Cre recombination via *Pcdh9-Cre* and *ChAT-Cre* allowed for specific conditional disruption and genetic access for viral tracing. *Pcdh9-Cre* is expressed in all RGC populations and many amacrine, bipolar, and horizontal cells throughout development; however, its expression resolves to superior oDSGCs and ON-SACs post-natally.^23,45^ In the brain, *Pcdh9-Cre* expression is restricted to RGC axon terminals, allowing for retina-specific disruptions with no expression in AOS downstream cells and expression only in other non-visual regions such as the olfactory bulb.^45^ In the retina, *ChAT-Cre* is solely expressed in SAC populations, beginning neonatally and persisting to adulthood.^46^

**Figure 2.**
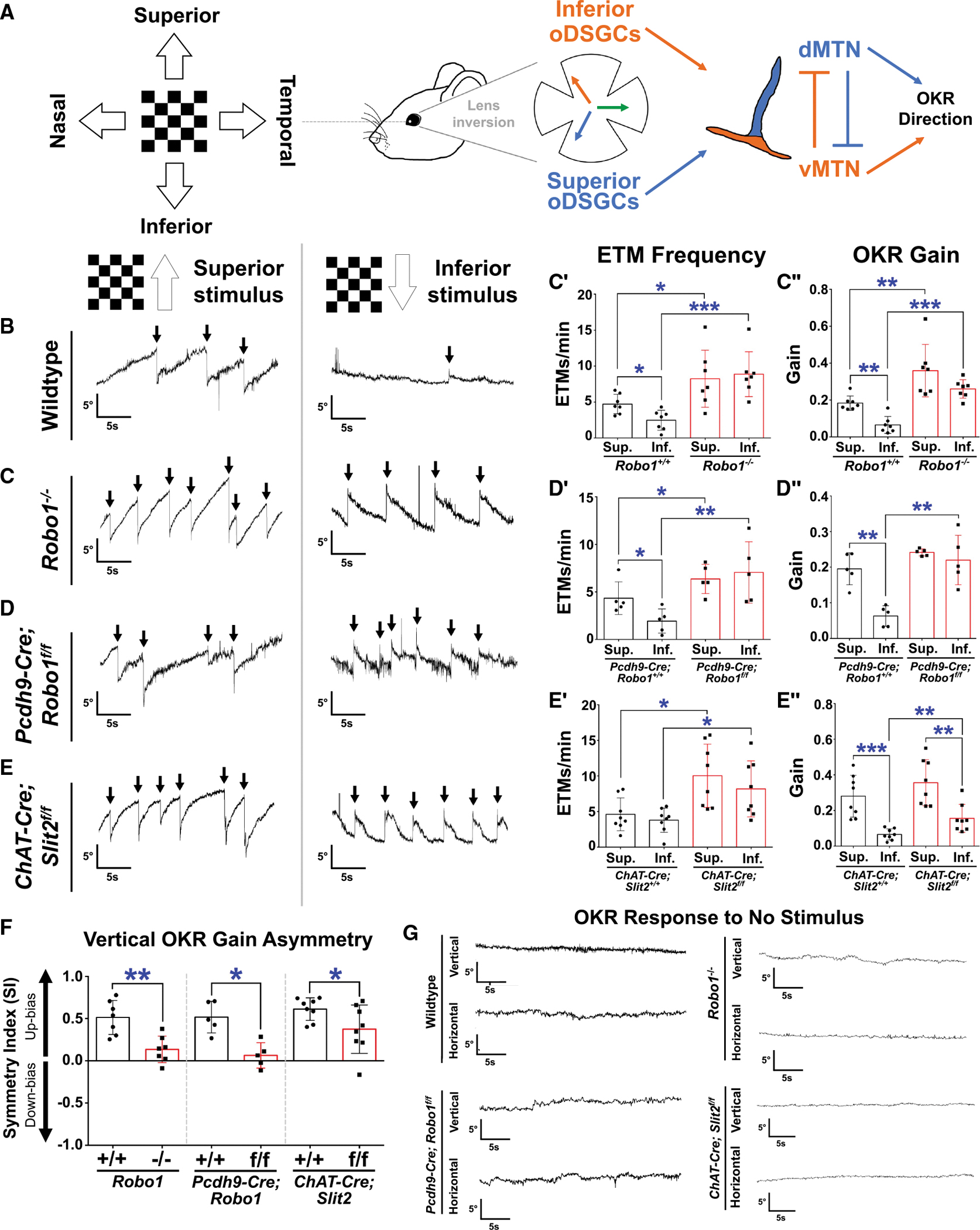
Vertical OKR responses in *Robo1* or *Slit2* mutants are enhanced and more symmetric (A) Diagram depicting the OKR circuit, from visual stimulation to oDSGCs to brainstem activation (MTN, medial terminal nucleus). (B–E) Superior and inferior OKR responses in wild type (B), *Robo1*^−/−^ (C), *Pcdh9-Cre; Robo1*^*f/f*^ (D), and *ChAT-Cre; Slit2*^*f/f*^ (E). Frequency of compensatory saccades (arrows) or eye tracking movements (ETMs) and OKR gain are increased between littermate controls and each mutant (C–C″, D–D″, and E–E″). (F) Vertical OKR asymmetry between superior and inferior OKR responses is reduced in all mutants, becoming more symmetric rather than superior-biased. (G) *Robo1* and *Slit2* LOF mutants do not exhibit uncontrolled eye movements in the absence of stimulation. *p < 0.05, **p < 0.01, ***p < 0.001. Bar graphs and error bars indicate the mean and SD. See also Figure S2.

We recorded OKR responses from each mutant line and littermate controls to unidirectional, continuously moving, high-contrast checkerboard gratings in the four cardinal directions at a speed of 5°/s (Figure 2A). All three mutants exhibited significant increases in both saccadic frequency and slow phase tracking gain^47^ in response to slow motion stimuli in some, if not all, four directions (Figures 2B–2E and S2A–S2D). Notably, the behavioral asymmetry conserved across species between superior (upward) and inferior (downward) motion is significantly reduced in all mutants, leading to a more symmetric vertical OKR (Figure 2F). This results in significant enhancement of inferior responses that increase the inferior OKR to levels similar to the superior OKR (Figure 2B), which normally greatly exceeds the inferior OKR^9,23,48^ (Figure 2F). Despite enhanced image tracking, all mutants retain directionally precise OKR responses. With no stimulation, both *Slit2* and *Robo1* knockouts do not make aberrant saccades or uncontrolled eye movements (Figure 2G). When we analyzed “cross-coupled” eye movements (eye movements along the orthogonal axis), we found that *Slit2* and *Robo1* mutants do not exhibit cross-coupled responses for most directions; however, we observed that inferior responses exhibit a naso-temporal bias (Figures S2E–S2J). These results demonstrate that global and retinal loss of *Robo1*, or SAC-specific loss of *Slit2*, lead to enhancement of OKR tracking, with elevated tracking gains in each direction and a significant reduction of the superior-biased vertical OKR asymmetry observed in littermate controls.

### *Robo1* refines oDSGC axonal projections to the MTN

**Figure 3.**
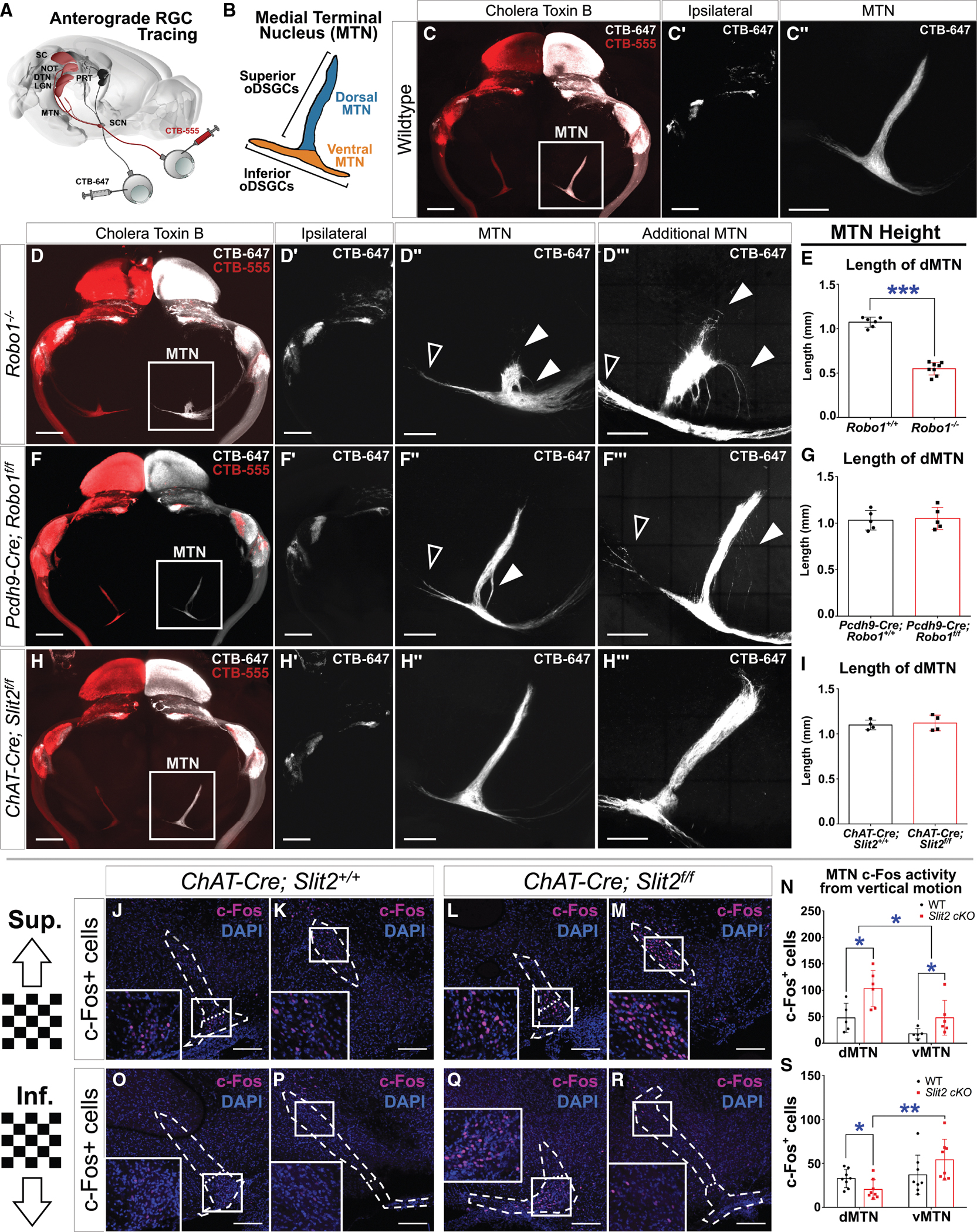
Robo1 is necessary to refine oDSGC axons, but loss of SAC-derived Slit2 reveals an additional function of modulating MTN responses upstream in the retina (A) Schematic of retinorecipient axonal labeling. (B) Anatomy of oDSGC projections within the MTN. (C) Maximum intensity projections of wild-type retinorecipient targeting. Insets: ipsilateral projections (C′) and contralateral MTN (C″). (D and E) *Robo1*^−/−^ animals exhibit MTN defects with curvature of dMTN-projecting axons (white arrows) and ectopic vMTN extension (black arrows) (D″ and D‴). The length of dorsally projecting axons in the dMTN is reduced (E). (F and G) *Pcdh9-Cre; Robo1*^*f/f*^ animals exhibit minor MTN defects with misinnervation of curving dMTN-projecting axons (white arrows) and ectopic extension in the vMTN (F″ and F‴). However, the length of the dMTN is not reduced (G). (H and I) *ChAT-Cre; Slit2*^*f/f*^ animals exhibit no MTN defects (H″ and H‴) and no change in dMTN length (I). All mutants exhibit normal ipsi- and contralateral segregation (D′, F′, and H′). (J–N) Assessment of c-Fos activation in the MTN in response to superior stimulation in both wild-type controls and *ChAT-Cre; Slit2*^*f/f*^ animals. Superior stimulation activates the vMTN (J and L) and dMTN (K and M) in both conditions. The dMTN in *Slit2* mutants exhibits significantly more activation than control dMTN (M). *Slit2* mutant MTNs have more c-Fos^+^ cells in the dMTN and vMTN than controls (N). (O–S) Inferior stimulation activates both the vMTN (O and Q) and dMTN (P and R) in both *ChAT-Cre; Slit2*^*f/f*^ and control animals. The vMTN in *Slit2* mutants exhibits significantly more activation than control vMTNs (Q). *Slit2* mutant MTNs have fewer c-Fos^+^ cells in the dMTN than controls and more cells in the vMTN compared with mutant dMTNs (S). Scale bars, 1 mm (C, C′, D, D′, F, F′, H, and H′), 500 μm (D″, D‴, F″, F‴, H″, and H‴), and 150 μm (J–M and O–R). *p < 0.05, **p < 0.01, ***p < 0.001. Bar graphs and error bars indicate the mean and SD. See also Figure S3.

Slit/Robo signaling drives RGC axon guidance.^26,27,29,36–38,49^ Since *Robo1* is expressed selectively in oDSGCs, we asked if Robo1 is necessary for oDSGC axonal projections to AOS nuclei. Using cholera toxin B (CTB) to label all RGC axons and their ipsilateral and contralateral projections (Figure 3A), we examined how RGC axon guidance to the MTN (Figure 3B) is affected in *Robo1* or *Slit2* mutants. Light-sheet imaging of cleared brains (STAR Methods) allowed for visualization of RGC axon targeting to retinorecipient targets.

Analysis of *Robo1* or *Slit2* mutant brains revealed varying MTN defects (Figures 3D–3I). In *Robo1*^−/−^ global null brains, the mutant MTNs appear severely disrupted, with a significant reduction of the dMTN, curvature of dMTN-projecting RGC axons, and ectopic medial extension of vMTN-projecting axons (Figures 3D and 3E). Despite the apparent reduction of the dMTN and disrupted MTN-projecting axons, *Robo1*^−/−^ global mutants retain the correct number of vertically tuned oDSGCs (Figures S3A–S3D) and also maintain downstream connectivity (Figures S3E–S3H). In *Pcdh9-Cre; Robo1*^*f/f*^ brains, the mutant MTNs exhibit a milder defect, with ectopic curvature of some dMTN-projecting axons and medial extension of a few vMTN-projecting axons; however, axonal extension into the dMTN is unaffected, unlike *Robo1*^−/−^ global null MTNs (Figures 3F and 3G). Sparse labeling of *Pcdh9-Cre*^+^ axons reveals that most superior oDSGC axons extend into the dMTN, but some ectopically target the vMTN (Figures S3I–S3N), suggesting the curvature phenotype arises from superior oDSGC axon mis-projection. Finally, in *ChAT-Cre; Slit2*^*f/f*^ brains, mutant MTNs exhibit no appreciable defects, with proper innervation of both dMTN and vMTN subnuclei as in wild-type controls (Figures 3H and 3I). In all mutants, other retinorecipient targeting remains grossly normal, as does ipsilateral and contralateral axonal segregation (Figures 3C–3H), as previously observed.^27^ Therefore, *Robo1* global function shapes oDSGC axon targeting within the MTN; however, Robo1 in RGCs plays a minor role in oDSGC MTN targeting.

### *Slit2* mutant MTN activation by select directional visual stimuli reflects behavioral increases in OKR responses

Despite no observable MTN defects, *ChAT-Cre; Slit2*^*f/f*^ animals still exhibit enhanced and more symmetric vertical OKR tracking (Figures 2E and 2F). Although *Pcdh9-Cre; Robo1*^*f/f*^ animals exhibit minor MTN defects, they retain significant OKR enhancements similar to *Slit2* mutants. Therefore, we asked whether the OKR enhancements are driven by retinal processing upstream of the MTN. In *Robo1* or *Slit2* mutants, OKR enhancement is observed in response to both superior and inferior motion. We assessed neuronal activity in the MTN when mice were subjected to vertical motion stimulation, leveraging immunofluorescence staining to detect the product of the activity-dependent gene *c-Fos* to assess activation of MTN neurons. In wild-type animals, upward stimulation preferentially activates the dMTN, and downward stimulation preferentially activates the vMTN, as reflected in c-Fos expression.^48,50^ However, downward stimulation elicits weaker MTN activity compared with upward stimulation, mirroring normal OKR behavioral asymmetry.^48^

Since MTN morphology was unchanged in our *ChAT-Cre; Slit2*^*f/f*^ animals, we investigated c-Fos activation in *ChAT-Cre; Slit2*^*f/f*^ MTNs in response to vertical stimulation. In the absence of stimulation, no c-Fos expression was observed in the MTNs of mutant or control animals (Figures S3X and S3Y). In the presence of superior stimulation, littermate controls exhibited increases in the number of MTN cells expressing c-Fos (Figures 3J and 3K) and in a proportion similar to that observed previously.^48^ In *ChAT-Cre; Slit2*^*f/f*^ mutants, we observed a significant alteration in visual stimulus-mediated activation of c-Fos expression (Figures 3L and 3M). We found that the number of c-Fos^+^ cells was significantly increased in *ChAT-Cre; Slit2*^*f/f*^ MTNs, particularly in the dMTN (Figure 3N). We next investigated MTN activity in response to inferior stimulation (Figures 3O–3S), observing a significant alteration in MTN c-Fos expression compared with wild type (Figures 3O and 3P), with increased activity in *ChAT-Cre; Slit2*^*f/f*^ animals particularly in the vMTN (Figure 3Q). The proportion of c-Fos^+^ cells that reside in the dMTN and the vMTN was shifted, with a significant decrease in the dMTN and an increase in the vMTN (Figure 3S). The dMTN and the vMTN primarily encode superior and inferior information, respectively, and these data show that the input of *Slit2* mutant superior and inferior oDSGCs to the MTN conveys a greater net directional vector in response to the preferred stimulus. These data also demonstrate that Slit2 functions in the retina to modulate information flow from oDSGCs to the midbrain and that disruption of this process likely leads to OKR enhancement.

Major MTN defects are present in *Robo1*^−/−^ global null mutants, and very mild MTN defects are observed in *Pcdh9-Cre; Robo1*^*f/f*^ animals. We performed MTN activation assays following the same paradigm using both mutant lines. In *Robo1*^−/−^ animals, we observed c-Fos^+^ cells within the MTN upon inferior stimulation; however, we were unable to accurately quantify them due to the severe MTN disruption in the global *Robo1*^−/−^ mutants (data not shown). In *Pcdh9-Cre; Robo1*^*f/f*^ animals, we observed a similar increase of vMTN activity as in *ChAT-Cre; Slit2*^*f/f*^ mutants, with an increased proportion of c-Fos activity in the vMTN following inferior motion stimulation (Figures S3O–S3W). Due to alterations in superior oDSGC projection (Figures S3I–S3N), we were only able to accurately assess responses to inferior stimulation. Taken together, these data provide evidence of a retina-specific modulating function by Robo1 similar to that observed in *Slit2* mutants.

### *Slit2* is necessary for refinement of excitatory synapses in oDSGC dendrites

Our results show that *Robo1* functions to guide MTN-projecting oDSGC axons and suggest Slit2/Robo1 signaling influences retinal processing to threshold OKR responses. To address Slit2/Robo1 retinal functions, we focused on *ChAT-Cre; Slit2*^*f/f*^ animals because the specificity of this disruption allows for direct linkage to observed oDSGC changes in OKR responses.

**Figure 4.**
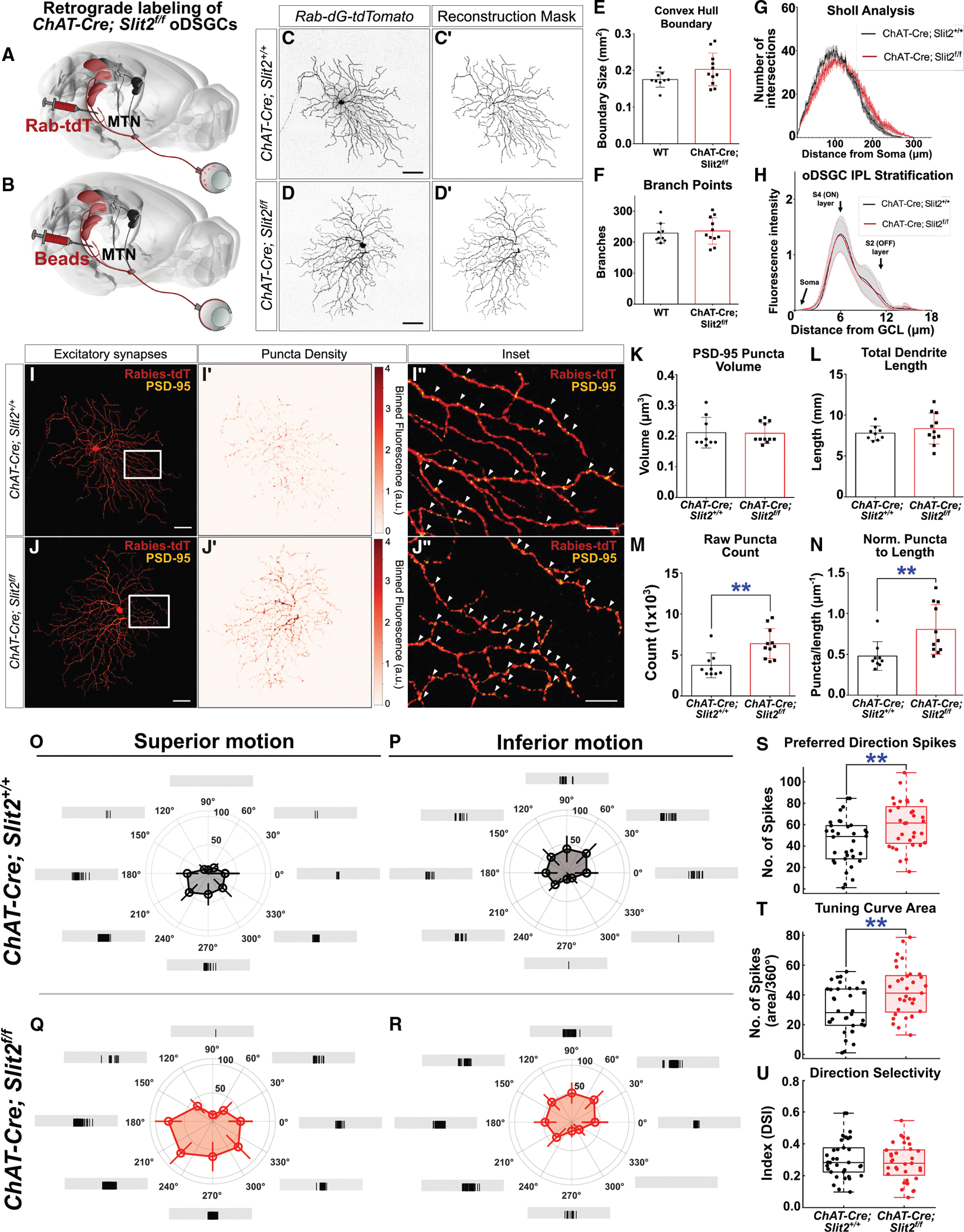
SAC-specific disruption of *Slit2* increases the number of excitatory synaptic puncta in MTN-projecting oDSGCs and enhances vertical oDSGC spike responses (A) Schematic of viral retrograde filling of MTN-projecting oDSGCs. (B) Schematic of fluorobead retrograde labeling of MTN-projecting vertical oDSGCs for electrophysiological recording. (C–H) *ChAT-Cre; Slit2*^*f/f*^ mutant vertical oDSGCs exhibit no gross morphological changes. *Slit2* mutant oDSGCs exhibit normal boundary sizes (E), branch points (F), complexity (G), and IPL stratification (H). (I and J) *Slit2*^*f/f*^*; ChAT-Cre* mutant oDSGCs exhibit an increase in excitatory puncta, as determined by immunolabeling of PSD-95 in masked oDSGC dendrites (I″ and J″) (arrows). A 2D histogram of puncta distribution (I′ and J′) reveals increased density in *Slit2* mutant dendritic arbors. (K–N) *ChAT-Cre; Slit2*^*f/f*^ mutant oDSGCs exhibit normal PSD-95 puncta volumes (K) and total dendritic length (L). However, they exhibit increased raw numbers of excitatory synaptic puncta (M) as well as normalized numbers of puncta per dendritic length (N). (O–U) Average tuning curve with sample spikes of superior and inferior oDSGC populations in both control (O and P) and *ChAT-Cre; Slit2*^*f/f*^ (Q and R) retinas. Coordinates are in retinal space. Both superior and inferior oDSGCs exhibit significant increases in spike responses to their preferred direction (S) and tuning curve area (T). Direction selectivity is retained in *Slit2* mutant oDSGCs, which display an unchanged direction selectivity index (U). Data were collected from 34 oDSGCs from 6 wild-type retinas and 33 oDSGCs from 5 *ChAT-Cre; Slit2*^*f/f*^ retinas. Scale bars, 50 μm (C, D, I, and J) and 15 μm (I″ and J″). **p < 0.01. Bars and error bars indicate the mean and SD (E, F, and K–N). Lines and shaded regions indicate mean and SEM (G and H). Circles and error bars indicate the mean and SD per direction (O–R). For box plots, horizontal line indicates the median, box boundaries indicate interquartile range (IQR), and whiskers indicate the most extreme observation within 1.5 × IQR. See also Figure S4.

We determine how vertical oDSGCs are affected in the absence of SAC-derived *Slit2* by assessing their dendritic morphologies and electrophysiological spike responses. We used stereotaxic injection of virally encoded retrograde tracers to fill oDSGCs targeting the MTN (Figure 4A) or to label them for electrophysiological recording (Figure 4B). Following skeletonization and reconstruction of individually filled oDSGC dendrites (STAR Methods), we found that *Slit2*^*f/f*^*; ChAT-Cre* mutant oDSGC dendritic arbors were grossly normal, with boundary sizes, branch points, and complexities similar to those observed in littermate controls (Figures 4C–4G). We observed no significant changes in dendritic laminar targeting within the retina; *Slit2* mutant oDSGC dendrites reside primarily in the S4 layer of the inner plexiform layer (IPL), with some minor variation in arbor localization that mirrors controls (Figure 4H).

We next asked whether *ChAT-Cre; Slit2*^*f/f*^ oDSGCs are more subtly disrupted. Slit/Robo synaptic regulatory functions have been linked to the control of excitatory synapse formation, with Robo1 functioning to balance excitatory inputs in hippocampal neurons^39^ and in bone cancer models.^51,52^ Given the increase in both OKR gain and c-Fos activity in the MTN of *ChAT-Cre; Slit2*^*f/f*^ mutants, we hypothesized that Slit2 refines excitatory synaptic inputs onto oDSGCs. We stained retrogradely filled oDSGCs for the excitatory postsynaptic density marker PSD-95 (Figures 4I and 4J) and assessed the abundance of PSD-95 puncta in *ChAT-Cre; Slit2*^*f/f*^ and control oDSGC dendritic arbors. Both superior and inferior oDSGCs were pooled for assessment. We observed normally sized excitatory puncta distributed in significantly greater numbers and densities in *Slit2* mutant oDSGC dendrites, suggesting an increase in excitatory inputs (Figures 4K–4N). Additionally, *Slit2* oDSGCs showed no significant changes in inhibitory synaptic puncta (Figures S4A–S4F) or gross changes in SAC dendritic morphology or lamination (Figures S4G–S4K), supporting the specificity of this disruption. Therefore, SAC-derived Slit2 does not regulate the formation of dendritic processes; however, SAC-derived Slit2 does suppress excitatory synapse formation and/or stabilization.

### Loss of SAC-derived *Slit2* enhances oDSGC spike responses

Since we observed a significant increase in excitatory synaptic puncta number in the absence of SAC-derived Slit2, we next asked if the spike responses of vertical oDSGCs in *ChAT-Cre; Slit2*^*f/f*^ mutant retinas are altered in response to vertical motion stimuli. Using labeled vertically tuned oDSGCs in *Slit2* mutant mice and littermate controls (Figure 4B), we conducted cell-attached recordings in retinal explants to measure oDSGC spiking in response to bars moving in 8 cardinal directions (STAR Methods).

Recording from MTN-projecting oDSGCs revealed expected superior and inferior vertical oDSGC populations in both mutant and control retinas (Figures 4O and 4P). In *ChAT-Cre; Slit2*^*f/f*^ retinas, we observed that both superior and inferior oDSGCs have greater spike responses and larger tuning curves compared with littermate controls (Figures 4Q and 4R). In *Slit2* mutant oDSGCs, the number of spikes elicited in their preferred direction (Figure 4S), and also in response to all directions (Figure 4T), was significantly greater; however, they still retained their direction selectivity to their preferred direction (Figure 4U). These results demonstrate that SAC-derived Slit2 modulates oDSGC spike responses. In its absence, oDSGCs spike more to directional stimulation, in line with our observations of increased excitatory puncta, increased c-Fos activity in the MTN, and overall increased OKR gains in *Slit2* SAC-specific loss-of-function (LOF) mutants.

## DISCUSSION

We have found a novel role for Slit2/Robo1 signaling in refining the activity of DS circuits necessary for controlling gaze stabilization behavior and tuning behavioral responses for naturalistic image movement. From cross-species and cross-temporal analyses of *Robo1* transcriptomic expression,^33,34,53^ we find that *Robo1* expression is highly conserved in oDSGCs in the ancient AOS system. *Slit2* or *Robo1* LOF releases a constraint in the AOS such that differences in the magnitudes of OKR responses to distinct directional stimuli are significantly altered. These include OKR enhancement to stimuli in all directions and a notable reduction in the normal asymmetry observed in the vertical OKR response. Robo1 regulates oDSGC axon guidance, likely through canonical Robo1 signaling, to refine MTN-projecting oDSGC axon targeting to the brainstem. In the absence of SAC-derived Slit2, downstream activity in the AOS significantly increases in response to directional stimulation, reflecting observed OKR enhancements. This is in line with increased excitatory synaptic puncta and enhanced spike responses of vertical oDSGCs. The apparent conservation of this tuning mechanism, revealed both in the retinal expression of *Robo1* and the presence of OKR behavioral asymmetries across phylogeny, suggests this Slit2/Robo1 constraint serves an important ethological function by generating regulated gaze stabilization, minimizing responses to less relevant stimuli like locomotive optic flow. These results demonstrate that Slit2/Robo1 signaling is essential for refining oDSGC circuit development and for controlling directional OKR responses necessary for image stabilization.

### Slit2/Robo1 signaling acts as a tuning mechanism to constrain gaze stabilization

Prioritization of relevant directional information is likely important in shaping gaze stabilization responses since both vertical and horizontal OKR and VOR asymmetries are preserved across most species.^1^ Among vertebrates, monocular posterior and also mono- and binocular inferior OKR responses are significantly weaker, despite differences in visual system diversity. These asymmetries have been proposed to offset directional information received from common sources, such as optic flow generated by forward locomotion^1,12^ or downward gravitational forces on the vestibular system.^10,11^ Forward locomotion primarily generates posterior and ventral optic flow, aligning with conserved OKR asymmetries.^54^ Since locomotive optic flow may be less critical and more distracting than other more relevant motion stimuli, OKR asymmetry likely evolved to minimize unnecessary eye or head movements through an intrinsic suppression mechanism.^9^ We identify Slit2/Robo1 signaling as a likely component of this tuning mechanism. In its absence, directional OKR responses remain intact; however, response magnitudes increase, with vertical OKRs becoming more symmetric. Since these alterations appear to arise from changes in oDSGC firing and the proportion of specific directional information conveyed through the MTN, we propose that Slit2/Robo1 signaling tunes retinal responses for the conveyance of directional information to the AOS. Loss of this tuning function reduces control over the magnitude of motion sensation, increases activation of downstream AOS neurons, and results in excessive eye movement due to unfiltered directional inputs.

### Loss of Slit2/Robo1 tuning releases directional suppression through increased oDSGC excitability

Increased responses from oDSGCs to AOS nuclei can explain how OKR tracking gains are enhanced by retinal circuitry. The magnitude of OKR gain is flexible and is influenced by interactions between the AOS and the visual cortex,^55^ the vestibular system,^5,18,56,57^ and the cerebellar flocculus,^56,58,59^ driving both long-term potentiation and acute enhancement of the OKR. Increased OKR gain, or optokinetic nystagmus (OKN) gain, has been primarily observed to result from the release of inhibition in the brain. In humans, primates, and mice, loss of graviceptive otolithic inputs through exposure to microgravity environments,^10,11,60,61^ sacculectomies,^62^ or mutations^57^ results in enhancement of vertical OKN responses and also loss of normal vertical asymmetry. In the case of reversible alterations, such as exposure to microgravity, OKN plasticity allows for a return to normal asymmetry upon return to standard gravity.^11,60,61^ This enhancement of OKN is attributed to the release of saccular inhibition, which suppresses the optokinetic system at normal gravity.^11^ Inhibition within the AOS also modulates OKR gain, and silencing of inhibitory NOT/DTN neurons increases horizontal OKR responses.^22^ Here, we report OKR gain increases originating from retinal AOS circuit components. Similar to how vestibular and brainstem inhibition shapes excitatory information in the AOS to control OKR output, oDSGC spikes dictate information flow into the AOS for generation of the OKR. When these sources of constraint are released, such as with loss of otolithic inputs or an increase of oDSGC firing, the AOS conveys increased responses, driving stronger OKR behavioral outputs.

The constant attenuation of directional input defined by Slit2/Robo1 signaling appears important for generating appropriate behavioral responses, reducing ethologically insignificant motion perception, and prioritizing important visual cues at the level of sensory input to the retina.

### Excitability of oDSGCs relies on balancing excitatory inputs through Slit2/Robo1 signaling

To our surprise, we found that disruption of Slit2/Robo1 signaling in oDSGCs impacts excitatory synapse number and does not alter major developmental processes, such as oDSGC dendritic elaboration or lamination. Our synaptic puncta assessments in oDSGC dendrites suggest that SAC-derived Slit2 is necessary to refine oDSGC responses by reducing excitatory transmission, likely through interactions with Robo1. Though we did not observe morphological changes in inhibitory puncta numbers, future studies will reveal how functional excitatory and inhibitory inputs to the oDSGCs change with disruption of Slit2/Robo1 signaling. Since *Robo1* is expressed primarily in oDSGCs among all RGCs and is not expressed in presynaptic ON bipolar cells,^63^ its cell-autonomous function in oDSGCs is likely responsible for refining excitatory synapses. However, greater temporal and cell-type specificity in *Robo1* disruption is needed to confirm its cell-autonomous role. Future analysis employing RGC-specific post-natal disruption of *Robo1* could confirm this cell-autonomous role in oDSGCs. Given the phenotypic specificity we observe in *ChAT-Cre; Slit2*^*f/f*^ animals despite the presence of Slit2 from other retinal neurons, localized and spatiotemporally regulated Slit2/Robo1 signaling between SACs and oDSGCs may modulate the proper level of oDSGC excitatory transmission. Future investigation will define the types of synapses involved, such as cholinergic vs. glutamatergic excitatory synapses, the subcellular distributions of Robo1 and Slit2 proteins, and any functional synaptic differences. Nevertheless, we show here that Slit/Robo signaling plays two distinct functions in shaping retinal and midbrain processes during oDSGC development.

### Slit2/Robo1 as a conserved tuning mechanism in the mammalian AOS

RGC transcriptomic profiling reveals that *Robo1* oDSGC-specific expression is phylogenetically conserved in the mammalian retina,^33,34^ from rodents to primates to humans. In humans, a *ROBO1* recessive missense variant has recently been linked to an isolated incidence of congenital nystagmus in which patients present solely with bilateral horizontal nystagmus and no other neurological symptoms.^64^ Since other pathologies, such as X-linked infantile nystagmus, have been linked to disrupted DS circuitry,^24^ loss of a similar ROBO1-mediated tuning mechanism could explain nystagmus pathology. *ROBO1* expression across mammalian oDSGC populations, along with the corresponding directional asymmetries in AOS-mediated image stabilization behaviors, suggests that the murine Slit2/Robo1 tuning mechanism we describe here functions more broadly to establish these asymmetries. The AOS is an ancient, highly conserved, primary visual system,^4,18^ and Slit2/Robo1-mediated mechanisms may have arisen in a common mammalian ancestor to tune oDSGC responses, warranting future investigation into how *ROBO1* functions in other visual systems.

Although our observations focus primarily on vertical motion, our data reveal that this mechanism functions in balancing horizontal motion responses as well. From our transcriptomic analyses, we find that *Robo1* is expressed selectively in all oDSGCs, including the putative forward oDSGC cluster. ON-SACs form connections with all oDSGC populations, and thus SAC-derived Slit2 could impact all oDSGC types. We observed similar OKR enhancements of horizontal responses in our *Robo1* and *Slit2* mutants. Crosstalk between horizontal and vertical axes is critical for generating proper OKR stabilization responses.^18,21,23,45,48^ However, since forward oDSGCs are currently inaccessible for precise genetic perturbation, future work will characterize how Silt2/Robo1 signaling functions in the horizontal axis and may mediate monocular horizontal OKR asymmetry.

*Robo1* and *Robo2* are the major Robo receptors expressed in the developing retina.^26–29,38,42^ *Robo2* plays key roles in regulating RGC axon targeting in the retina,^29^ the optic chiasm,^26,27^ and retinorecipient targets.^27^ Consequently, *Robo1* has been viewed as serving a redundant function in the visual system; however, we describe a unique role for Robo1 in regulating oDSGC refinement. Although Robo receptors can compensate for each other,^36^ we still observe a significant disruption of oDSGC formation in the absence of only *Robo1*, demonstrating a function not compensated for by *Robo2*. How *Robo2* impacts AOS function in *Robo1*^−/−^ oDSGCs remains to be determined.

Taken together, we describe here a retinal mechanism that attenuates directional information from sensory input so as to prioritize relevant visual stimuli. We find that Slit2/Robo1 signaling acts to regulate OKR responses by filtering visual information through repressive modulation of DS ganglion cell responses. These findings reveal how complex retinal circuits compute visual input and convey sensory information to instruct proper behavioral responses.

## RESOURCE AVAILABILITY

### Lead contact

Further information and requests for resources and reagents should be directed to and will be fulfilled by the lead contact, Alex L. Kolodkin (kolodkin@jhmi.edu).

### Materials availability

Strains generated for this study are available from the lead contact upon request.

### Data and code availability

- All data available in this paper will be shared by the lead contact upon request.
- All original code and processed data used in this paper have been deposited on Zenodo (Zenodo: https://doi.org/10.5281/zenodo.17065906) and are publicly available as of the date of publication.
- Any additional information required to reanalyze the data reported in this paper is available from the lead contact upon request.

## STAR★METHODS

### EXPERIMENTAL MODEL AND SUBJECT DETAILS

All animal experiments were approved by the Institutional Animal Care and Use Committee (IACUC) at The Johns Hopkins University School of Medicine and at UCSF. The day of birth was designated as postnatal day 0 (P0). Mice of either sex ranging in age from P5 to P90 were used. *Robo1*^−/−^ and *Slit2*^*flox*/+^ animals were a gift from L. Ma from Thomas Jefferson University and were described previously.^36,43^ *ChAT-Cre* mice (Stock #006410) were obtained from the Jackson Laboratory. *Robo1*^*flox/flox*^ animals were generated at The Johns Hopkins University School of Medicine Transgenic Core. All mice were maintained in a C57BL/6J background. Animals were housed in a 12-hour light-dark cycle. Behavioral tests were performed at consistent hours during the light cycle.

### METHOD DETAILS

#### Intraocular CTB and AAV injections

Mice were anesthetized with 2% isoflurane. A hole was made at the corneal limbus with a 30 G needle and 1 μl of CTB, AAV2, or AAV9 was injected intravitreally using a Hamilton syringe. For co-injections, 0.75 μl of CTB with 0.75 μl of AAV2 were co-injected. Injected animals were sacrificed two days post injection for CTB labeling. AAV injected animals were sacrificed two weeks post injection.

#### Brain clearing and lightsheet imaging

For brain clearing and imaging of retinorecipient targeting, CTB-injected animals were transcardially perfused with phosphate buffered saline (PBS) with 0.5% heparin added to reduce residual blood coagulation, followed by perfusion with 4% paraformaldehyde (PFA). Extracted brain was fixed in 4% PFA overnight at 4°C, then washed 3 times with PBS for 30 minutes each to remove residual PFA. Brains were then cleared. Brains were dehydrated in 20%, 40%, 60%, and 80% methanol (MeOH) in H_2_O at room temperature (RT) for 40 minutes for each dilution. They were then washed twice in 100% MeOH for 40 minutes each, and then stored overnight at 4°C. Following dehydration, they were then washed in a 66% dichloromethane (DCM)/33% MeOH solution for 3 hours at RT. They were then washed twice in 100% DCM at RT for 15 minutes each before being placed into dibenzyl ether (DBE) overnight. The following morning, DBE was replaced with fresh DBE and left to sit for around 8 hours, or until the brains had significantly cleared. DBE was then changed to ethyl cinnamate (ECi) overnight for imaging. For imaging, cleared brains were imaged on a Zeiss Lightsheet 7 microscope with a 5x objective in ECi with a refractive index of 1.558.

#### Stereotaxic surgery and MTN injections

Adult mice were anesthetized using 2% isoflurane and placed into a stereotaxic apparatus. Lumafluor-555 retrobeads or Rabies virus were injected into the medial terminal nucleus (600 nL at 10 nL/s and 400 nL at 10 nL/s respectively) using a Hamilton Neuros syringe via an automatic microsyringe controller (World Precision Instruments). The MTN was injected at the following coordinates relative to bregma: anterior/posterior 0 mm, medial/lateral ± 0.85 mm, dorsal/ventral −5.4mm with a needle tilt of 30° anteriorly. Needle was brought to lowest dorsal/ventral depth, raised 0.2mm, and injected. Following injection, five minutes were allowed to elapse before removing the syringe to allow for diffusion to occur. For bead injection, the surgery was only performed on the right hemisphere. For rabies virus injection, the surgery was performed on both hemispheres.

#### Immunohistochemistry

Immunohistochemistry staining was performed for whole-mount retina staining and cross-sectional brain staining according to protocol as previously described.^23^

Primary antibodies used in this study include: Rabbit anti-dsRed (Living Colors, 1:1000), Mouse anti-PSD95 (Abcam, 1:500), Rabbit anti-cFos (CST, 1:1000).

#### Fluorescent in situ hybridization

Fluorescent *in situ* hybridization experiments were performed on fixed and fresh frozen retinas in both whole-mount and cryosections respectively. For whole-mounts, mice were anesthetized with 2% isoflurane and decapitated. Enucleated eyes were then fixed in PFA overnight at 4°C, and then dissected to remove the retina and whole-mount it on a slide for staining. For cryosections, mice were anesthetized with 2% isoflurane and decapitated. Enucleated eyes were immediately embedded in Neg-50 frozen section medium, frozen on dry ice, and sliced with a cryostat at a thickness of 15μm. Fluorescent *in situ* hybridization were then performed on both whole-mount or cryosectioned retinas using the Hybridization Chain Reaction RNA-FISH Technology from Molecular Instruments according to the standard protocol. Probes for *Robo1*, *Slit2*, *Tbx5*, *Fibcd1*, and *ChAT* were obtained from the manufacturer, which was generated by the manufacturer from full length RNA sequences for each gene. Dotted lines were drawn on cells of interest to mark areas of mRNA puncta clusters for genes of interest. For binning quantification, cells with >5 puncta were manually counted and considered as positive for the gene of interest.

#### Headpost implantation surgery

Adult mice (≥P41) were anesthetized with 2% isoflurane and underwent headpost implantation as described previously.^23^

#### Optokinetic reflex recording

Headfixed mice were placed into a custom animal holder and placed into a square box surrounded with 4 monitors to cover the visual field of the headfixed animal. Visual stimuli were presented, and the optokinetic reflex responses were recorded through the same method as described previously.^23^ To elicit the reflex response, unidirectional stimuli of black and white checkerboard (rotation speed: 5° of visual angle/s; stimulus cycle: 30 s of stimulus, followed by 30 s of grey; 15 cycles per direction) were presented to the mouse continuously rotating in either upward, downward, forward, and backward directions. Recording of eye movements and of presented stimuli were custom-built in Matlab and LabView as described previously.^23^

Processed data were analyzed using Igor Pro v6.37 (WaveMetrics, Portland OR) as well as Python package PyOKR. Compensatory eye tracking movements were manually counted in Igor Pro as a metric that has been described previously. Slow phase tracking gains were quantified from continuous tracking waves by analysis via PyOKR as described previously.^47^

#### Assessment of c-Fos activity in the MTN

To assess c-Fos activation within relevant brain regions, intraocular injections of CTB were performed in animals ≥2 days prior to stimulation in order to label retinorecipient regions. Prior to stimulation, animals were dark-adapted for ≥2 hours before being subjected to continuous directional stimulation for 1 hour. Mice were immediately sacrificed and perfused. Brain tissue was then fixed in 4% PFA for 1 hour at room temperature. Fixed brains were then sectioned on a vibratome at a thickness of 150μm and stained against c-Fos. For quantification, c-Fos^+^ cells were identified by co-staining with DAPI and counted based on localization positive cells within MTN-projecting CTB axons. Total counts were quantified through addition of all sections containing MTN-projecting CTB signal so as to include all c-Fos^+^ cells within the MTN. Distinction of dorsal vs. ventral MTN was defined at a 170μm cutoff, with CTB labeling above 170μm from the bottom of the MTN defined as dMTN and CTB labeling below defined as vMTN.

#### Electrophysiology

As described above, vertically preferring oDSGCs were identified by retrobead labeling from the MTN. To ensure that retrobeads could be visualized in oDSGCs during the recording, electrophysiology experiments were conducted at least 2 days following injections of retrobeads into the MTN. Mice were dark-adapted overnight and euthanized under infrared illumination by cervical dislocation prior to recording. Because retrograde tracers were injected into the MTN on the right hemisphere, the contralateral (left) eye was enucleated. Retina dissections were performed in the dark using infrared converters on a dissecting microscope and whole-mounted on a glass coverslip. During the dissection and throughout the recording experiment, the retina was perfused with warmed, oxygenated (95% O_2_/5% CO_2_) Ames solution. Following enucleation, brains were harvested and fixed in 4% PFA. Brains were then sectioned using a vibratome and imaged to confirm that the retrograde tracer was injected into the MTN.

Cell-attached recordings of oDSGCs were performed using two-photon targeting as described previously.^8^ Briefly, retrogradely-labeled retinal ganglion cells were identified using a two-photon microscope (peak emission at 860 nm). Using IR light to visualize the tissue, cells were recorded using patch electrodes (Sutter) filled with HEPES buffered Ames. Data was acquired at 10kHz with a MultiClamp 700B amplifier using Symphony 2 DAS, run through Matlab software. The projector was centered on the soma of the cell and covered a 427 × 311 μm region. Cell responses were recorded to bars (3.2 degrees in width) moving at 10 degrees/sec in 8 directions (separated by 45 degrees). For each cell, five repetitions of each stimulus direction were collected and the order of presentation for each direction was randomized. The moving bar stimuli were generated using Stage and presented via a DLP projector (peak emission at 405 nm).

Analysis of all recordings was performed in Matlab and responses were averaged across 5 trials per stimulus direction. Preferred directions for each cell were calculated as the direction of the vector sum of spike responses to all stimulus directions. The direction selectivity index (DSI) was calculated as the vector sum of responses divided by the scalar sum of responses to all stimulus directions.

#### Dendritic morphology analysis

To label vertical oDSGC dendritic arbors, stereotaxic injections to the MTN were performed (as described above) in adult (≥P41) animals with 400nL of rabies virus expressing either mCherry (Rab-dG-CAG-mCherry) or tdTomato (Rab-dG-CAG-tdT) to retrogradely label oDSGC dendritic arbors in the retina. After 3 days, injected animals were sacrificed, perfused with 4% PFA for 30 minutes at RT, and placed in PBS. Retinas were then dissected and stained with immunohistochemistry to enhance the viral fluorescence (Rb anti-DsRed) and label excitatory synapses (Ms anti-PSD95). Retinas were then whole-mounted, and sparsely labeled oDSGC arbors were imaged at high resolution.

Following image collection, oDSGC arbors were traced via Simple Neurite Tracer (SNT) in ImageJ. From the skeletonization of the arbors, measurements were recorded including: convex hull boundary size, Sholl analysis, number of branch points, and total dendrite length as described in built-in functions.

To quantify synaptic puncta, the arbor skeletonization was used to generate a binary mask with the “Fill Out” function in SNT. The binary mask was then used in ObjectFinder,^65^ a MatLab-based application, to identify colocalized puncta objects within the dendritic arbor. The binary mask was applied to PSD-95 staining of the same image, and objects were identified with the following parameters: diameter of object: 0.4μm-3μm and minimum object intensity greater than 2x the background intensity. From the report, puncta volume and number of puncta were recorded to quantify amounts of PSD-95 expression within a single arbor. Fluorescence curves of oDSGC arbor depths across the IPL were quantified with a custom Python script.

### QUANTIFICATION AND STATISTICAL ANALYSIS

Single-cell RNAseq clustering, UMAP plots, dotplots, and violin expression plots were generated with Scanpy.^66^ Volcano plots were generated with Scanpy differential gene expression analysis and visualized with a custom Python script. Symmetry Index was calculated $SI=\frac{{\mathrm{Gain}}_{up}\;-\;{\mathrm{Gain}}_{\mathrm{down}}}{{\mathrm{Gain}}_{up}\;+\;{\mathrm{Gain}}_{\mathrm{down}}}$. Reconstruction of retinal mosaic cell locations and WGA+ cell distributions were identified using CellProfiler and analyzed through custom Python scripts. Nearest Neighbor calculations were performed with the OPIPP Python package.^67^ Dendritic reconstructions were generated through SNT in ImageJ, and associated puncta counting was performed via ObjectFinder. Additional graphs used for quantification were generated using GraphPad Prism. Mean comparisons were performed with Wilcoxon Rank Sum Tests. Distribution comparisons were performed with Kolmogorov–Smirnov tests. Sholl analysis was performed with a two-way ANOVA.

#### Single-cell RNA sequencing data analysis

Single-cell RNA sequencing analysis was performed on previously generated datasets to profile: P5 RGCs,^30^ P4–12 oDSGCs,^23^ adult RGCs across 15 mammalian species,^33^ and human fetal RGCs.^34^

Processed reads were accessed under GEO number GSE115404 and analyzed as an AnnData object in Scanpy in Python v3.10.1. Cluster 32 was then sub-clustered with unsupervised leiden clustering, and visualized as a UMAP of 3 distinct subclusters. Differential gene expression analysis and comparison to known markers was performed to confirm the identity of these three clusters as different oDSGC populations. Visualization of UMAP projections and dot plot expression were generated through built-in plotting functions in Scanpy v1.10.3.

The P4–12 On DSGC dataset was analyzed from processed reads accessed under GEO number GSE211344 and analyzed as an AnnData object in Scanpy v1.10.3 in Python v3.10.1. Unsupervised leiden clustering was performed to separate superior and inferior oDSGC populations across time. Gene expression levels of Cluster 32, superior, and inferior oDSGC markers were visualized as violin plots and dot plots using built-in functions in Scanpy v1.10.3.

An integrated RGC atlas profiling 15 mammalian species was accessed via GEO number GSE237215, with integration, plotting, and analysis R scripts generated by the Shekhar lab (https://github.com/shekharlab/RetinaEvolution) but modified to visualize oDSGC and *Robo1* conservation across mammalian RGCs.

The human fetal RGC dataset was analyzed from processed reads accessed under GEO number GSE268630 and analyzed as an AnnData object in Scanpy v1.10.3 in Python v3.10.1. RGC populations were separated from other retinal subtypes computationally, integrated across developmental time, and clustered with unsupervised leiden clustering. Cross-referencing of murine, primate, and human oDSGC marker genes were used to determine the oDSGC cluster. Differential gene expression was performed with Scanpy v1.10.3 and built-in plotting functions were used to plot UMAP and dot plots. Volcano plot of putative oDSGC markers was generated using a custom Python script.

## Supplementary Material

1

SUPPLEMENTAL INFORMATION

Supplemental information can be found online at https://doi.org/10.1016/j.cub.2025.08.030.

## Figures and Tables

**KEY RESOURCES TABLE:** 

REAGENT or RESOURCE	SOURCE	IDENTIFIER

Antibodies		

Rabbit polyclonal anti-DsRed	Living Colors	Cat # 632496; RRID: AB_10013483
Mouse monoclonal anti-PSD-95 MAGUK scaffolding protein	Neuromab	Cat # 75-028; RRID: AB_2292909
Mouse monoclonal anti-Gephyrin	Synaptic Systems	Cat # 147111; RRID: AB_887719
Rabbit monoclonal anti-cFos	Cell Signaling Technology	Cat # 2250; RRID: AB_2247211

Bacterial and virus strains		

AAV2 CAG-mWGA-mCherry (2E+12)	Virovek, Inc.	Cat # 117BUCSF89
AAV9-EF1a-BbChT	Addgene	Cat # 45186
SAD-B19-RVdG-tdTomato (3.50E+10)	UC Irvine Vector Core	Cat # RVdG-5

Chemicals, peptides, and recombinant proteins		

DAPI (4’,6-Diamidino-2-Phenylindole, Dihydrochloride)	Life Technologies	Cat # D1306; RRID: AB_2629482
Cholera Toxin Subunit B (Recombinant), Alexa Fluor 555 Conjugate	Thermo Fisher	Cat # C34776
Cholera Toxin Subunit B (Recombinant), Alexa Fluor 647 Conjugate	Thermo Fisher	Cat # C34778
Lumafluor Red Retrobeads	Lumafluor, Inc.	https://lumafluor.com/
Ames’ Medium	US Biological	Cat # A1372-25

Critical commercial assays		

HCR RNA-FISH Kit (v3.0)	Molecular Instruments	https://www.molecularinstruments.com/
HCR Probe - Robo1-B5	Molecular Instruments	https://www.molecularinstruments.com/
HCR Probe - Tbx5-B3	Molecular Instruments	https://www.molecularinstruments.com/
HCR Probe - Fibcd1-B4	Molecular Instruments	https://www.molecularinstruments.com/
HCR Probe - Slit2-B5	Molecular Instruments	https://www.molecularinstruments.com/
HCR Probe - ChAT-B4	Molecular Instruments	https://www.molecularinstruments.com/

Experimental models: Organisms/strains		

Mouse: *Robo1*^−/−^	Gift from L. Ma^36^	N/A
Mouse: *Robo1^flox/flox^*	This study	N/A
Mouse: Tg(Pcdh9-cre)NP276Gsat/Mmucd	MMRRC	Stock # 036084-UCD; RRID: MMRRC_036084-UCD
Mouse: *Slit2^flox/+^*	Gift from L. Ma^43^	N/A
Mouse: B6;129S6-Chat^*tm2(cre)Lowl*^/J	Jackson Laboratory	Stock # 006410; RRID:IMSR_JAX:006410

Oligonucleotides		

Robo1^flox/flox^ Forward Primer: CAGAGCGTACATGCTTGCTG	Integrated DNA Technologies	N/A
Robo1^flox/flox^ Reverse Primer: TGGAAATGGCCTAAGCATCA	Integrated DNA Technologies	N/A

Software and algorithms		

Python version 3.10.1	Python Software Foundation	RRID: SCR_008394
ImageJ	NIH	RRID: SCR_003070
Igor Pro version 6.37	WaveMetrics	RRID: SCR_000325
MATLAB	MathWorks	RRID: SCR_001622
LabVIEW 2012 version 12.0f3	National Instruments	RRID: SCR_014325
CellProfiler	CellProfiler Image Analysis Software	RRID:SCR_007358
ScanImage	MBF Bioscience	https://www.mbfbioscience.com/products/scanimage
StreamPix	NorPix	https://www.norpix.com/products/streampix/streampix.php
Symphony and Stage	Github	https://github.com/Symphony-DAS/symphony-matlab; https://github.com/Stage-VSS/stage-v1
ObjectFinder	Della Santina et al.^65^	https://github.com/lucadellasantina/ObjectFinder; https://zenodo.org/record/4767847; RRID:SCR_023319
Custom analysis code	This paper	Github: https://github.com/JKiraly/Slit2-Robo1-Project; Zenodo: https://doi.org/10.5281/zenodo.17065906

## Highlights

- *Robo1* or *Slit2* loss increases optokinetic reflex gain and vertical symmetry
- *Robo1* refines direction-selective ganglion cell axons within central targets
- *Slit2* or *Robo1* retinal loss drives enhanced central accessory optic system activity
- *Slit2* loss in presynaptic neurons increases excitation to direction-selective cells
