# Supplementary material for: Slit2/Robo1 signaling constrains image stabilization responses to preserve ethologically favorable directional asymmetry
Source: Curr Biol. Author manuscript; Available in PMC 2025 Oct 25. (PMC12553481; doi:10.1016/j.cub.2025.08.030)
Supplement: 1 [file NIHMS2116086-supplement-1.pdf]

Current Biology, Volume *35*

Supplemental Information

Slit2/Robo1 signaling constrains image stabilization responses to preserve ethologically favorable directional asymmetry

James K. Kiraly, Annika Balraj, Paige Leary, Zihao You, Scott C. Harris, Jeanette D. Hyer, Felice A. Dunn, and Alex L. Kolodkin

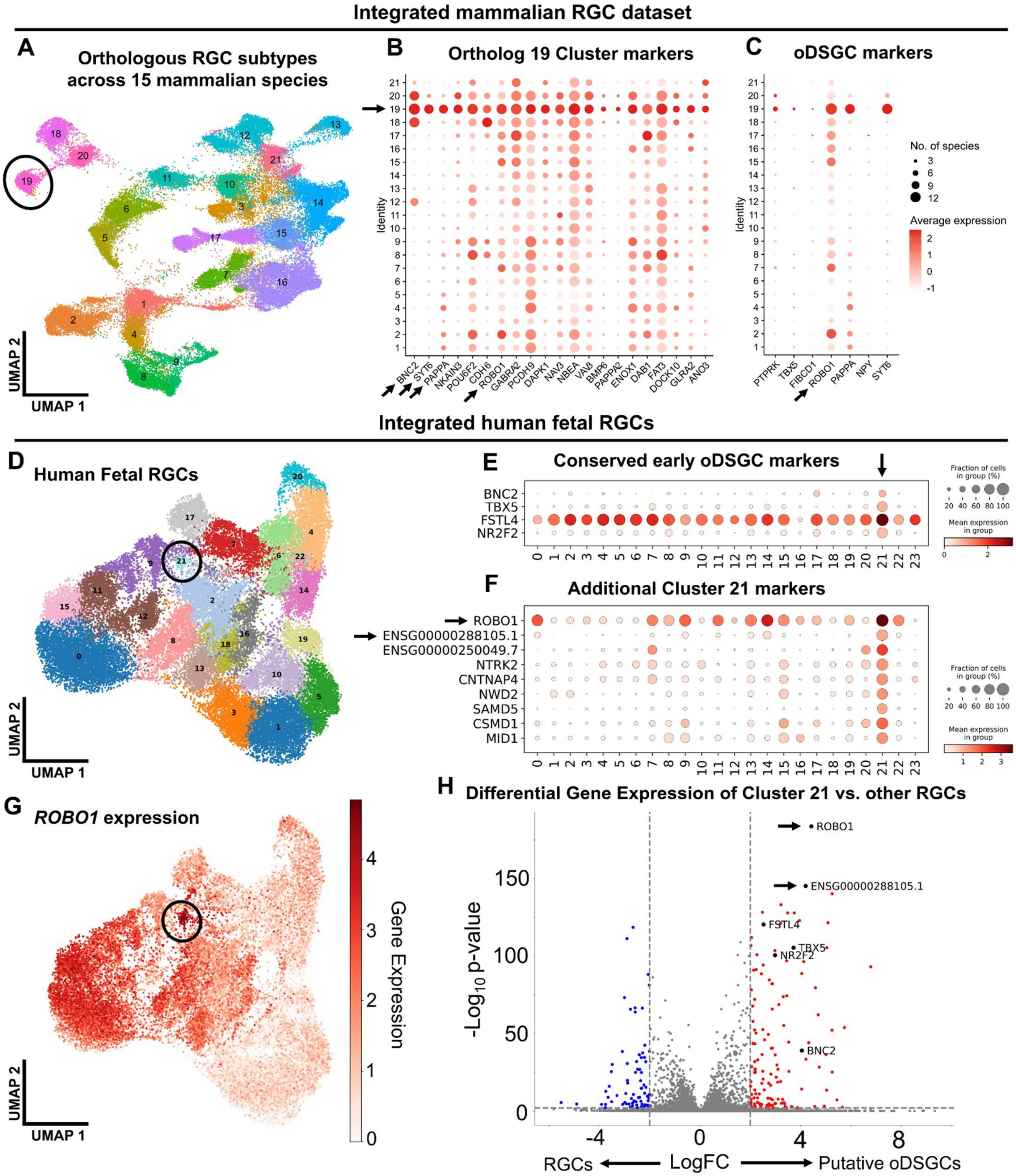
Integrated mammalian RGC dataset
A
Orthologous RGC subtypes across 15 mammalian species
UMAP 2
UMAP 1
B
Ortholog 19 Cluster markers
Identity
BNC2
SYT6
PAPPA
NKAIN3
POU6F2
CDH6
ROBO1
GABRA2
PCDH9
DAPK1
NAV3
NBEA
VAV3
BMP6
PAPPA2
ENOX1
DAB1
FAT3
DOCK10
GLRA2
ANO3
C
oDSGC markers
PTPRK
TBX5
FIBCD1
ROBO1
PAPPA
NPY
SYT6
No. of species
Average expression
Integrated human fetal RGCs
D
Human Fetal RGCs
E
Conserved early oDSGC markers
BNC2
TBX5
FSTL4
NR2F2
Fraction of cells in group (%)
Mean expression in group
F
Additional Cluster 21 markers
ROBO1
ENSG00000288105.1
ENSG00000250049.7
NTRK2
CNTNAP4
NWD2
SAMD5
CSMD1
MID1
G
ROBO1 expression
Gene Expression
H
Differential Gene Expression of Cluster 21 vs. other RGCs
FSTL4
TBX5
NR2F2
BNC2
-Log10 p-value
RGCs
LogFC
Putative oDSGCs

Figure S1: Specificity of *Robo1* expression is conserved across the mammalian retina, related to Figure 1.

(A) UMAP showing orthologous RGC clusters, as described by Hahn and colleagues (2023). AOS oDSGCs reside in orthologous cluster 19. **(B)** Dotplot depicting unsupervised marker genes of orthologous cluster 19. Note *ROBO1* appears as a highly conserved oDSGC marker across most mammalian species, along with *BNC2*, *SYT6*, and *PAPPA* (arrows). **(C)** Dotplot of murine vertical oDSGC markers shows the level of specificity to cluster 19, but varying degrees of conservation. **(D)** UMAP plot of human fetal RGC clusters integrated across developmental time. Putative oDSGCs reside in Cluster 21. **(E)** Dotplot of oDSGC markers shows specificity to Cluster 21 (arrow). **(F)** Dotplot of Cluster 21 differentially expressed marker genes reveals high differential expression of *ROBO1* and novel *ROBO1*-associated lncRNA (arrows) in Cluster 21. **(G)** *ROBO1* is highly and differentially enriched in Cluster 21 cells (circle). **(H)** Volcano plot of Cluster 21 differentially expressed genes with oDSGC markers displayed. *ROBO1* and its novel lncRNA are highly enriched and differentially expressed (arrows).

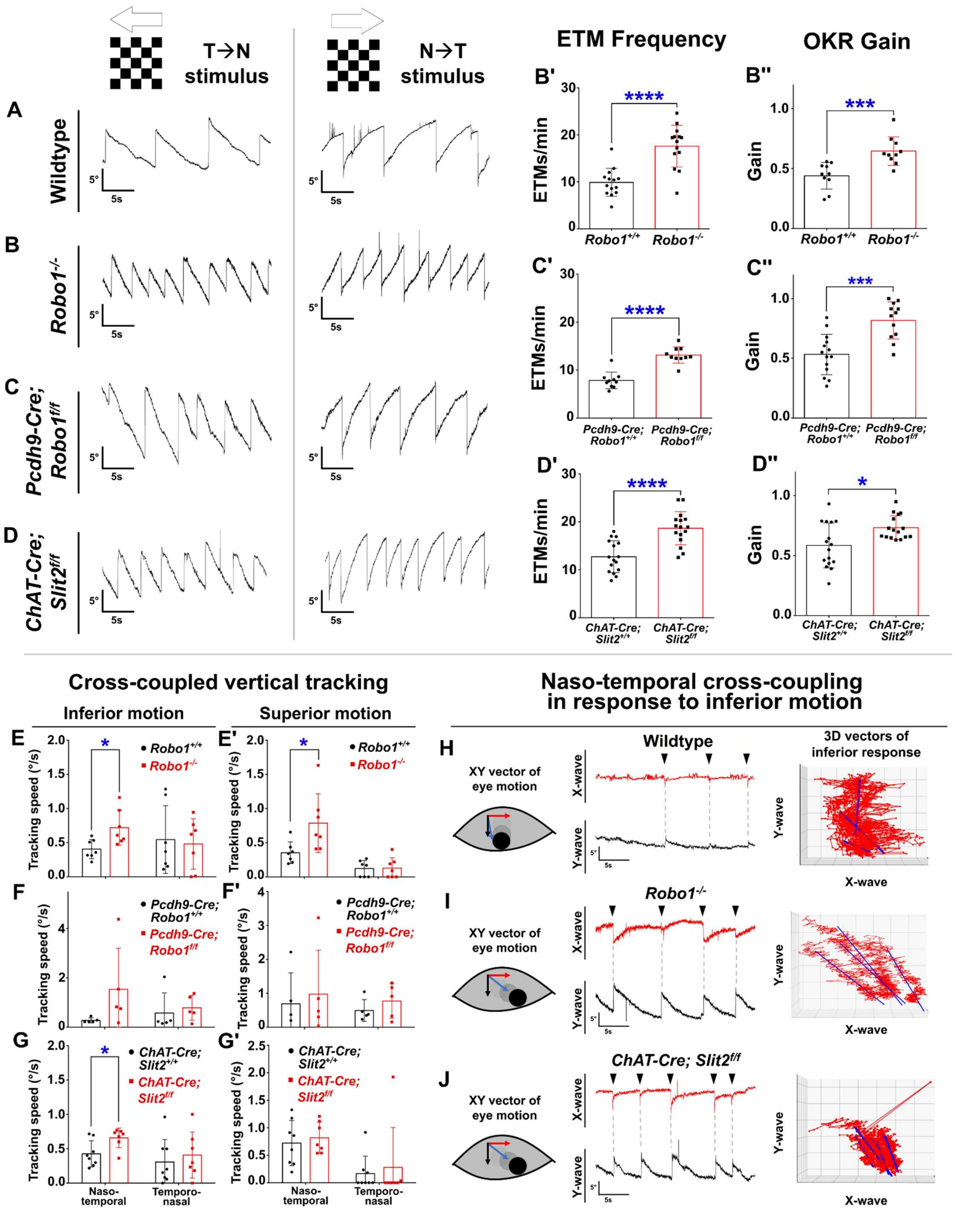

T→N stimulus
N→T stimulus
ETM Frequency
OKR Gain
A Wildtype
B Robo1-/-
C Pcdh9-Cre; Robo1f/f
D ChAT-Cre; Slit2f/f
5°
5s
B' C' D'
ETMs/min
B'' C'' D''
Gain
Robo1+/+
Robo1-/-
Pcdh9-Cre; Robo1+/+
Pcdh9-Cre; Robo1f/f
ChAT-Cre; Slit2+/+
ChAT-Cre; Slit2f/f
Cross-coupled vertical tracking
Inferior motion
Superior motion
E E' F F' G G'
Tracking speed (°/s)
Naso-temporal
Temporo-nasal
Naso-temporal cross-coupling in response to inferior motion
H Wildtype
I Robo1-/-
J ChAT-Cre; Slit2f/f
XY vector of eye motion
X-wave
Y-wave
3D vectors of inferior response

Figure S2: *Robo1* and *Slit2* mutants exhibit enhanced horizontal OKR responses and cross-coupled responses to inferior motion, related to Figure 2.

(A-D) Combined temporo-nasal and naso-temporal OKR responses in Wildtype (A), *Robo1*$^{-/-}$ (B), *Pcdh9-Cre; Robo1*$^{f/f}$ (C), and *ChAT-Cre; Slit2*$^{f/f}$ (D). ETM frequency and OKR gains are significantly enhanced in both temporo-nasal and naso-temporal directions (B'-B'', C'-C'', D'-D''). **(E-G)** Average horizontal eye movement in response to inferior or superior motion. Naso-temporal cross-coupling is observed in response to inferior stimulation; also observed in *Robo1*$^{-/-}$ superior response (E'). **(H-J)** Examples of naso-temporal cross-coupling in response to inferior stimulation. *p<0.05, **p<0.01.

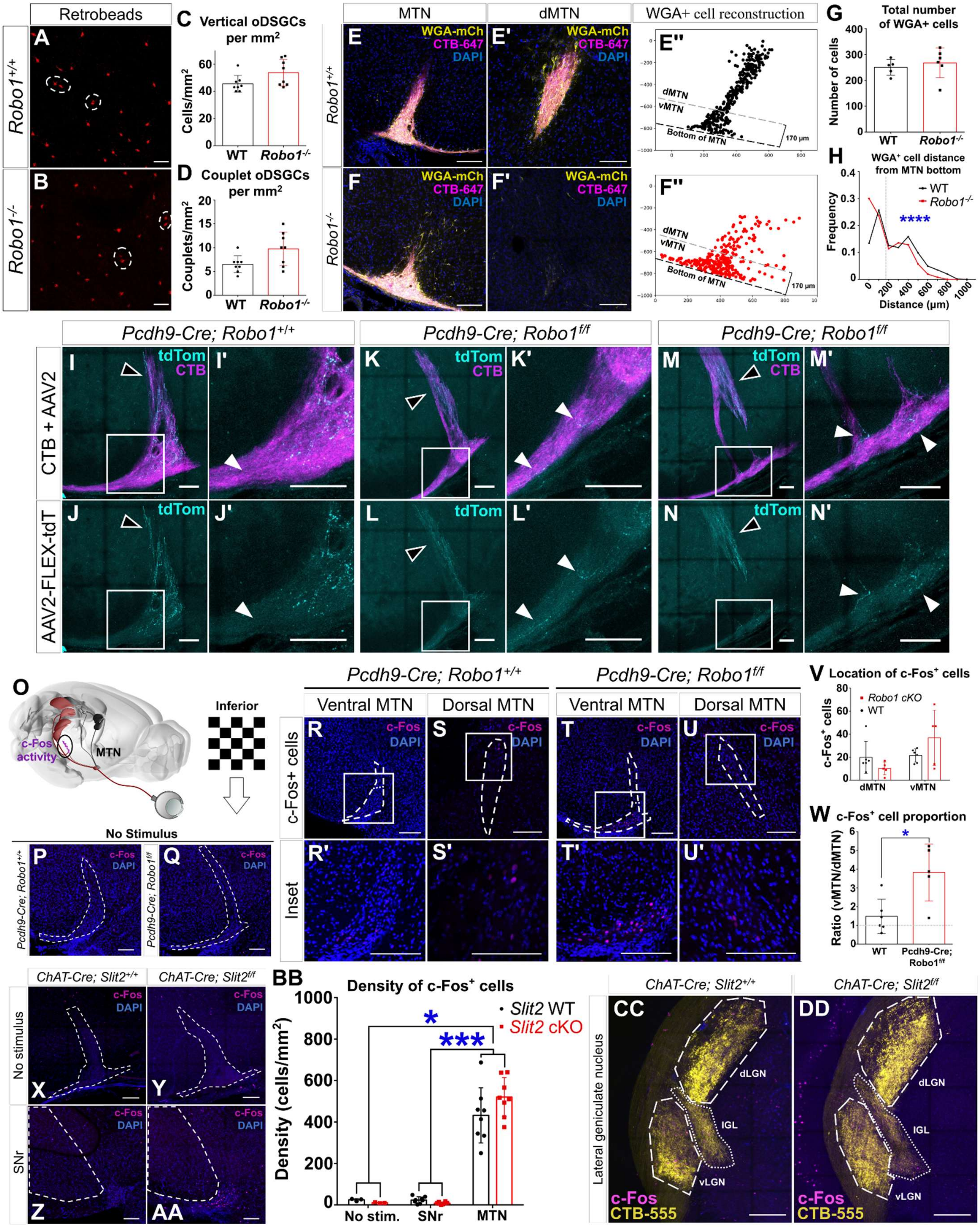
Retrobeads
Robo1+/+
Robo1-/-
A
B
C Vertical oDSGCs per mm2
Cells/mm2
WT
Robo1-/-
D Couplet oDSGCs per mm2
Couplets/mm2
MTN
dMTN
WGA+ cell reconstruction
E
E'
E''
F
F'
F''
WGA-mCh
CTB-647
DAPI
dMTN
vMTN
Bottom of MTN
170 μm
G Total number of WGA+ cells
Number of cells
H WGA+ cell distance from MTN bottom
Frequency
Distance (μm)
****
Pcdh9-Cre; Robo1+/+
Pcdh9-Cre; Robo1f/f
CTB + AAV2
AAV2-FLEX-tdT
tdTom
CTB
I
I'
J
J'
K
K'
L
L'
M
M'
N
N'
O
c-Fos activity
MTN
Inferior
No Stimulus
P
Q
c-Fos
DAPI
Ventral MTN
Dorsal MTN
c-Fos+ cells
Inset
R
S
T
U
R'
S'
T'
U'
V Location of c-Fos+ cells
c-Fos+ cells
Robo1 cKO
WT
dMTN
vMTN
W c-Fos+ cell proportion
Ratio (vMTN/dMTN)
WT
Pcdh9-Cre; Robo1f/f
*
ChAT-Cre; Slit2+/+
ChAT-Cre; Slit2f/f
No stimulus
SNr
X
Y
Z
AA
BB
Density of c-Fos+ cells
Density (cells/mm2)
Slit2 WT
Slit2 cKO
*
***
No stim.
SNr
MTN
CC
DD
Lateral geniculate nucleus
dLGN
IGL
vLGN
c-Fos
CTB-555

Figure S3: *Robo1$^{-/-}$* make downstream connections in the MTN and *Pcdh9-Cre; Robo1$^{f/f}$* mutants exhibit increased MTN activity similar to *Slit2* mutants, related to Figure 3.

(A-D) Retrobead MTN backfills to label MTN-projecting vertical oDSGCs in *Robo1$^{-/-}$* and control retinas (A-B). Global loss of *Robo1* does not significantly reduce the density of MTN-projecting oDSGCs (C-D). **(E-H)** Transsynaptic viral labeling shows that *Robo1$^{-/-}$* MTN-projecting oDSGCs make proper connections (E-F) with quantification (E'', F'') showing similar numbers of WGA^{+} cells (G), though a shift of cell location ventrally (H). **(I-N)** Sparse labeling of Superior oDSGC axons in *Pcdh9-Cre; Robo1$^{f/f}$* MTNs compared to control. In wildtype MTNs, most Superior oDSGCs extend to the dMTN (black arrow), with no innervation of the vMTN (white arrow) (I-J). In *Pcdh9-Cre; Robo1f/f* MTNs, most Superior oDSGCs extend to the dMTN (black arrows), but some axons extend deep within the vMTN (white arrows) (K-L) as well as along the ectopic projection from the dMTN (white arrows) (M-N). **(O)** Schematic of MTN activation by inferior stimulation. **(P-Q)** *Pcdh9-Cre; Robo1$^{f/f}$* and control animal MTNs do not activate in the absence of visual stimulation. **(R-W)** Inferior stimulation activates both the vMTN (R, T) and the dMTN (S, U) in both groups of animals. Activation in the vMTN is elevated in response to inferior stimulation (T'). Though overall changes in c-Fos^{+} cell location are not observed (V), normalized proportions of vMTN versus dMTN c-Fos^{+} cells shows a proportional increase of vMTN activity compared to controls (W). **(X-BB)** Negative controls for *Slit2* c-Fos experiments. In the absence of stimulus, the MTN does not exhibit c-Fos activity in either wildtype or *Slit2* mutants (X-Y). The neighboring region, the substantia nigra pars reticularis (SNr), does not exhibit any activation during motion stimulation (Z-AA). Quantification of c-Fos^{+} cell density reveals specific activation of the MTN in the presence of motion stimulation (BB). **(CC-DD)** Positive control for retinorecipient activity. The lateral genicular nucleus (LGN) shows similar patterns of c-Fos activity in both mutant and control, with high activity in the vLGN and IGL but little in the dLGN. Scale bars: 300μm (A-B), 250μm (CC-DD), 150μm (E-F, E'-F', P-U, and X-AA), and 75μm (I-N). *p<0.05, ***p<0.001 ****p<0.0001.

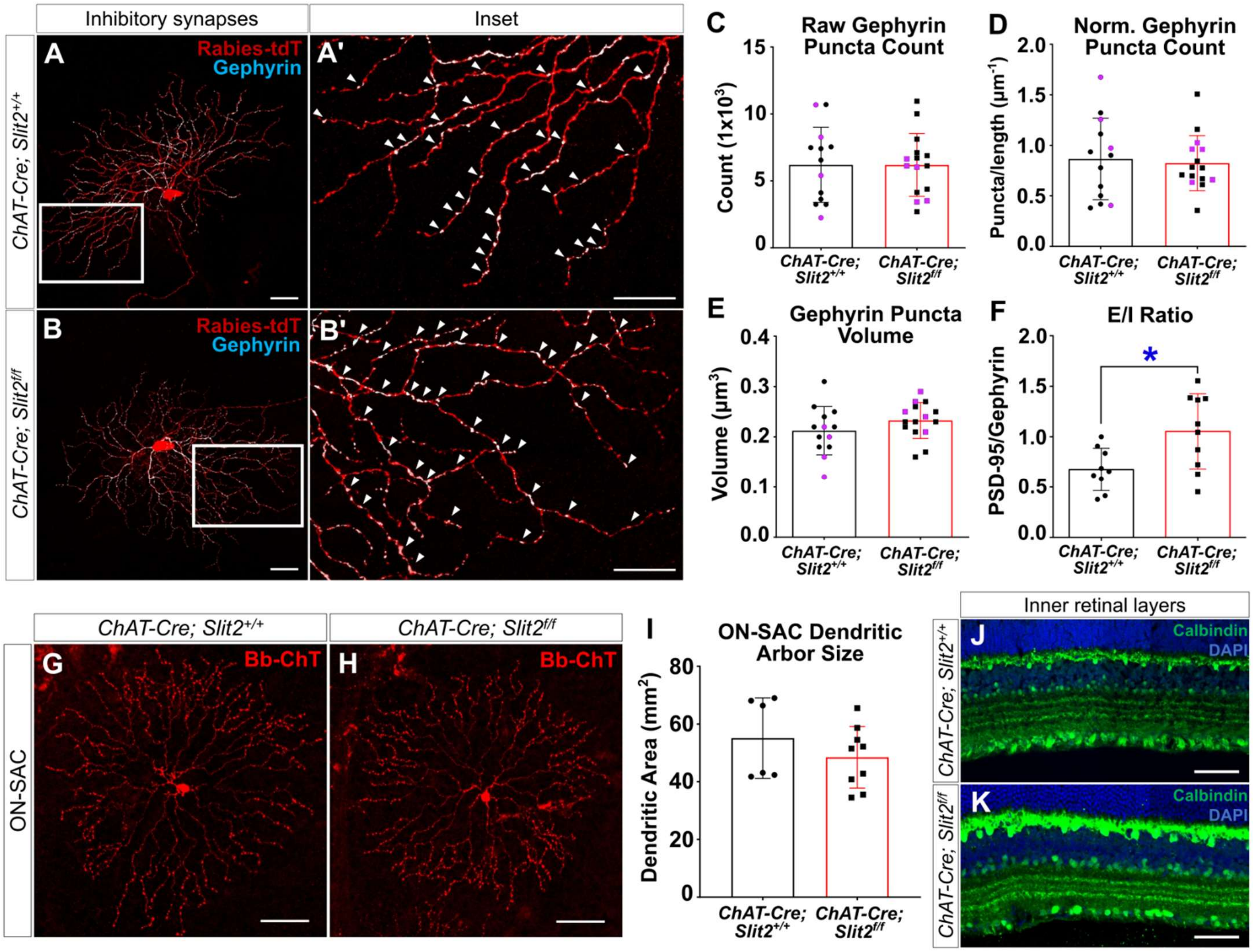

Inhibitory synapses
Inset
ChAT-Cre; Slit2+/+
ChAT-Cre; Slit2f/f
A
Rabies-tdT
Gephyrin
A'
B
B'
C
Raw Gephyrin Puncta Count
Count (1x10^3)
D
Norm. Gephyrin Puncta Count
Puncta/length (µm^-1)
E
Gephyrin Puncta Volume
Volume (µm^3)
F
E/I Ratio
PSD-95/Gephyrin
*
ChAT-Cre; Slit2+/+
ChAT-Cre; Slit2f/f
ON-SAC
G
H
Bb-ChT
I
ON-SAC Dendritic Arbor Size
Dendritic Area (mm^2)
Inner retinal layers
J
K
Calbindin
DAPI

Figure S4: Loss of *Slit2* does not impact inhibitory synaptic formation or gross IPL morphology, related to Figure 4.

(A-F) Vertical oDSGCs imaged from Figure 4 do not exhibit significant alterations in postsynaptic gephyrin densities. Masking of mutant and control dendrites for immunofluorescent staining of gephyrin reveals inhibitory synaptic puncta (A-B). No significant alteration in raw puncta count (C), normalized puncta count by dendritic length (D), or puncta volume (E) is observed. Points in magenta were collected from filled oDSGC arbors following electrophysiological recording. Comparison of excitatory puncta vs. inhibitory puncta within each cell reveals an expected shift towards excitation (F). **(G-I)** Loss of SAC-derived Slit2 does not grossly impact ON-SAC morphology (G-H). Quantification of dendritic arbors reveals no significant change in SAC size (I). **(J-K)** Lamination of the IPL is not disrupted in *ChAT-Cre; Slit2*$^{f/f}$ retinas, with S2-4 layers observable in both wildtypes (J) and mutants (K). Scale bars: 50µm (A-B, G-H, and J-K) and 15µm (A'-B'). *$p<0.05$.
